# Impact of multi-professional, scenario-based training on postpartum hemorrhage in Tanzania: a quasi-experimental, pre- vs. post-intervention study

**DOI:** 10.1186/s12884-017-1478-2

**Published:** 2017-09-05

**Authors:** Signe Egenberg, Gileard Masenga, Lars Edvin Bru, Torbjørn Moe Eggebø, Cecilia Mushi, Deodatus Massay, Pål Øian

**Affiliations:** 1 Department of Obstetrics and Gynecology, Stavanger University Hospital, Gerd-Ragna Bloch Thorsens gate 8, 4011 Stavanger, Norway; 2 Department of Obstetrics and Gynecology, Kilimanjaro Christian Medical Centre, Moshi, Tanzania; 3 Center for Behavioral Research, University of Stavanger, Stavanger, Norway; 4 National Center for Fetal Medicine, Trondheim University Hospital, Trondheim, Norway; 5 Mbulu, Manyara Tanzania; 6 Department of Obstetrics and Gynecology, University Hospital of North Norway, Tromsø, Norway; 7 Department of Clinical Medicine, University of Tromsø, Tromsø, Norway

**Keywords:** Multi-professional, Scenario-based training, Postpartum hemorrhage, Teamwork, Blood transfusion, Debriefing

## Abstract

**Background:**

Tanzania has a relatively high maternal mortality ratio of 410 per 100,000 live births. Severe postpartum hemorrhage (PPH) is a major cause of maternal deaths, but in most cases, it is preventable. However, most pregnant women that develop PPH, have no known risk factors. Therefore, preventive measures must be offered to all pregnant women.

This study investigated the effects of multi-professional, scenario-based training on the prevention and management of PPH at a Tanzanian zonal consultant hospital. We hypothesized that scenario-based training could contribute to improved competence on PPH-management, which would result in improved team efficiency and patient outcome.

**Methods:**

This quasi-experimental, pre-vs. post-interventional study involved on-site multi-professional, scenario-based PPH training, conducted in a two-week period in October 2013 and another 2 weeks in November 2014. Training teams included nurses, midwives, doctors, and medical attendants in the Department of Obstetrics and Gynecology. After technical skill training on the birthing simulator MamaNatalie®, the teams practiced in realistic scenarios on PPH. Each scenario was followed by debriefing and repeated scenario. Afterwards, the group swapped roles and the observers became the participants.

To evaluate the effects of training, we measured patient outcomes by determining blood transfusion rates. Patient data were collected by randomly sampling Medical birth registry files from the pre-training and post-training study periods (*n* = 1667 and 1641 files, respectively). Data were analyzed with the Chi-square test, Mann-Whitney U-test, and binary logistic regression.

**Results:**

The random patient samples (*n* = 3308) showed that, compared to pre-training, post-training patients had a 47% drop in whole blood transfusion rates and significant increases in cesarean section rates, birth weights, and vacuum deliveries. The logistic regression analysis showed that transfusion rates were significantly associated with the time period (pre- vs. post-training), cesarean section, patients tranferred from other hospitals, maternal age, and female genital mutilation and cutting.

**Conclusions:**

We found that multi-professional, scenario-based training was associated with a significant, 47% reduction in whole blood transfusion rates. These results suggested that training that included all levels of maternity staff, repeated sessions with realistic scenarios, and debriefing may have contributed to reduced blood transfusion rates in this high-risk maternity setting.

## Background

There is an inequity regarding access to skilled birth attendance, due to the fact that 78% of the world’s pregnant women have access to less than 42% of the world’s midwives, nurses and doctors [1]. According to “The state of the world’s midwifery 2014”, 73 countries carried 96% of the global burden of maternal mortality. Additionally, countries with low resources are challenged by “the three delays”: delay in seeking care, delay in reaching a health facility, and delay in receiving proper care at the facility [2, 3]. Tanzania has had insufficient progress related to maternal health, based on the Millennium Development Goal 5 [4]. The latest figures indicated that the Tanzanian maternal mortality ratio was 410 deaths per 100,000 live births, and the new 2030 target is 140 maternal deaths per 100,000 live births [4]. One major problem is the shortage of skilled birth attendants [2]. In 2012, the general availability of midwifery in Tanzania was estimated to fulfill only 74% of the need [1].

Postpartum hemorrhage (PPH) is most commonly defined as blood loss >500 ml after vaginal delivery and >1000 ml after cesarean section (CS) [5, 6], based mainly on visual estimation of blood loss. The figures on PPH-incidence vary from below 1% to 32%, depending on populations studied and definitions used [7–17]. World Health Organization reported in 2012 that PPH affects 2% of childbirths [18].

Because two-thirds of pregnant women that develop PPH have no known risk factors, preventive measures must be offered to all pregnant women [19, 20], including active management of the third stage of labor [21]. The most effective interventions that contribute to saving maternal lives are skilled birth attendants and emergency obstetric care [4].

Severe PPH is one of the most important causes of maternal deaths, and can cause death within hours. Causes of PPH can be described as related to tone (70–80%), tissue, trauma and/or thrombin. Factors that increase the risk for PPH are previous CS [22], previous PPH [7, 23], anemia [7, 23], malaria [24], severe preeclampsia in current pregnancy [23] and pregnancy-induced hypertension [14]. Known risk factors related to uterine atony are multiple pregnancies [7, 20, 23], large for gestational age [7, 14, 20], labor induction and augmentation [7, 20, 22], prolonged labor [7, 14, 20], and full urine bladder [20]. Retained placental tissue [20] and abnormal invasive placenta [7, 20, 22] can cause PPH, as well as trauma, like lacerations of the genital tract, episiotomy, female genital mutilation and surgery on the uterus [20, 22]. Various coagulopathies are uncommon but serious causes of PPH [7, 20].

This on-site, pre- vs. post-interventional study purposed a context-specific approach which involved all maternity staff, and implemented realistic scenarios. Consecutive debriefings challenged each participant on their frame of understanding within this multi-professional team. We hypothesized that this training would contribute to positive consequences for team efficiency and patient outcomes. The primary aim of this study was to determine whether this multi-professional, scenario-based training in managing PPH was associated with changes in blood transfusion rates.

## Methods

### Study setting

Kilimanjaro Christian Medical Centre (KCMC) is a zonal consultant hospital in the northern zone of Tanzania, with a catchment of 15 million people. KCMC provides care for 500–800 inpatients daily and a birth cohort of approximately 4000 mothers/year. Out of a total of 1300 staff members at KCMC, the Department of Obstetrics and Gynecology has approximately 85 nurses, midwives, doctors, and medical attendants. These providers are allocated to the labor ward and postnatal wards, which in addition to all deliveries and postnatal care for mother and child, treat women with complicated pregnancies and gynecological diseases. Additionally, the labor ward has surgery facilities. Many of the staff members at KCMC had previously participated in health provider training courses like Comprehensive emergency obstetric and newborn care (CEmONC) and/or Advanced life support in obstetrics (ALSO). However, this study provided the first training experience, where small, multi-professional teams that comprised all members of the maternity staff within the same facility trained together and discussed their team performance.

### Study design

#### Overall design

This study had a quasi-experimental, pre- vs. post-interventional design. Training on PPH was provided in an on-site, scenario-based, 6-h program for a multi-professional team of 8–10 employees. The training program comprising the entire maternity staff, was carried out during 2 weeks in October 2013 and repeated during 2 weeks in November 2014, organized by the same faculty. The effects of this maternity staff training were measured by comparing patient outcomes before and after training. We retrieved data on patient outcomes from patient files, 12 months before the training (2012) and 12 months (November 2013 to October 2014) after the first training period.

#### The training faculty

The training faculty was five senior Tanzanian midwifery leaders at KCMC, recruited by the management. They remained as faculty throughout the study. Two of them engaged as facilitators for the scenario and debriefing sessions and two others acted as laboring women by operating the birthing simulator, MamaNatalie [25]. Also, one faculty midwife who coordinated the training, was responsible for establishing multi-professional teams of 8–10 nurses, midwives, doctors, and medical attendants. Helping mothers survive: Bleeding after birth (BAB) learning materials [26] were translated into Swahili by the research team for this project.

A two-day preparatory course was conducted for the facilitators, operators, and the research coordinator. This course was facilitated by a senior consultant at KCMC who had been responsible for ALSO courses in Africa. The preparatory course included discussions on best practices, review of the KCMC guidelines for diagnosis and management of PPH [5], and review of the graphic BAB learning material, based on WHO guidelines for PPH [27]. The KCMC guidelines for PPH diagnosis and management during pregnancy and childbirth elaborated on the four T’s that cause PPH: tone, trauma, tissue, and thrombin [5]. The BAB learning package corresponded to the PPH procedures established at the KCMC. The participants shared and discussed relevant articles on human error, how to facilitate debriefing, and the quality of debriefing with good judgment [28–31]. The training focused on technical skills and non-technical skills like communication, including to call for help, decision-making, leadership, respectful care and information to patient and relatives. These issues were emphasized by the facilitator in the briefing session, challenged in the scenarios, and discussed during the debriefing sessions after the scenarios for reflective learning.

#### Intervention: maternity staff training

To ensure scientific basis, the simulation training was to an extent carried out in line with the evidence based «golden rules» of the PROMPT-courses: multi-professional participants, team training in local context, use of patient actors and debriefing of participants after the scenarios [32]. The training was strengthened by newly validated learning materials [33] in Swahili. Additionally, repeated scenarios as in this study was in accordance with Kolb’s experiential learning theory, explaining how knowledge can be created through exposure to concrete experiences, followed by reflective learning, abstraction of the concept and by applying new frames of understanding during a second experience [34]. To master any procedure, it is important to repeat the scenario and observe colleagues vicariously [35] to gain a new understanding [34] and to reframe one’s perception, which enhances self-efficacy [36]. To accomplish this, on the day of training, each team was divided into two small teams of 4–5 trainees. During the day, the two teams swapped places, to experience both scenario-participation and observation.

**Fig. 1:**
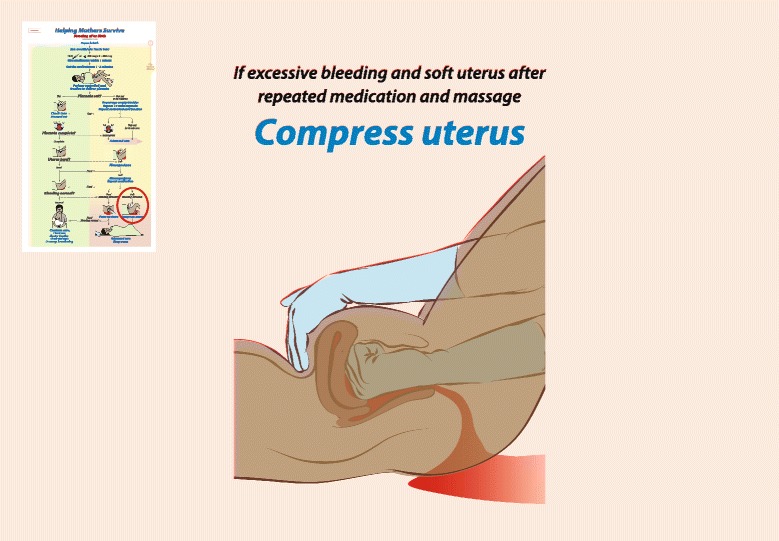
Bimanual uterine compression displayed in the Helping Mothers Survive pamphlet, provided in the BAB learning materials from Jhpiego: Jhpiego.Helping Mothers Survive [www.helpingmotherssurvive.org]

The facilitator presented the PPH procedure to the participants with the BAB graphic flipbook and took time to discuss the best practices. In particular, the diagram of bimanual compression was referred to throughout the training. The basic features of MamaNatalie included the baby manikin, NeoNatalie®, the placenta, uterus, a blood tank containing 1500 ml artificial blood, and a urine bladder. These items were demonstrated during a scenario on a normal delivery. Hands-on training enabled the trainees to practice uterine massage and bimanual uterine compression before the scenario-based training. Each participant received a Provider’s guide booklet on PPH, *Kuwanusuru Wakina Mama* (Helping mothers survive) [37], enclosed the image of bimanual compression, for further reading after the training (Fig. 1).

The facilitators and operators prepared an easily recognizable case to serve as the scenario on PPH. In this scenario, PPH was caused by uterine atony, the most common cause of PPH. The trainees were given clinical, and recognizable, information on the mother in the scenario; «Para 6, full-term. PPH at her last delivery. A spontaneous vaginal delivery 15 min ago after 12 h with inadequate contractions. Baby girl 4 kg. Placenta not yet born, normal bleeding, oxytocin given i.m.».

**Fig. 2:**
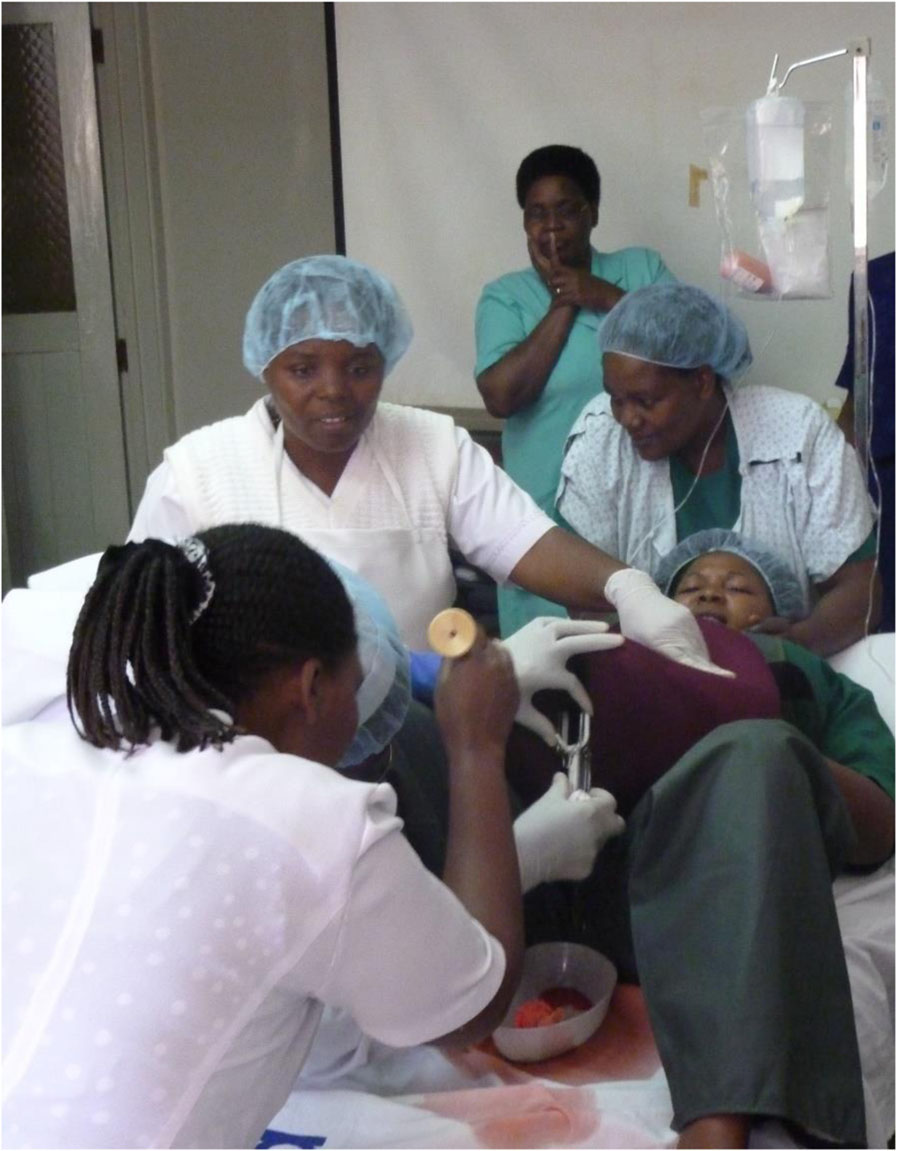
Multi-professional scenario-based training on PPH at KCMC

The learning goals for the training were: prevent PPH, identify and treat PPH, and communicate adequately. The operator made the scenarios realistic by enabling the participants to engage with the mother character, which demanded proper actions and communication. Staffing, equipment, and medicines in the scenario training reflected the typical situation in the wards. The scenario ended when the team controlled the bleeding or decided to transfer the patient to an operating theater for further treatment (Fig. 2).

The learning environment focused on critical thinking more than pure memorization; therefore, it was perceived as psychologically safe [38]. After every PPH scenario, the facilitator led the debriefing session with common reflection. Every trainee was challenged on their frames of understanding, perceived achievements, and possible future actions within a team. The debriefing session aimed to elicit honest feedback. Also the operator, a trainee acting as her relative, and the observers gave feedback on the service and the state of communication; here, the unique perspectives of all participants were valued [31]. The scenario was repeated and again debriefed. Afterwards, the entire group swapped roles and the observers became the participants in a similar scenario. They then debriefed and repeated the exercise, as described above. Thus, a total of four PPH-scenarios were conducted for the entire group throughout the day of training.

#### Approvals

The study at KCMC was approved and renewed on a yearly basis, locally and nationally by: (1) The Institute of Research Board, Kilimanjaro Christian Medical College Research Ethics and Review Committee; research proposal no. 504, clearance certificate no. 537. (2) The National Institute for Medical Research; ref. NIMR/HQ/R.8a/Vol.IX/1550. (3) The Tanzania Commission for Science and Technology; research permit no.2013–245-ER-2013-112. In addition, an ethics permit was granted from the Regional Ethics committee, Norway, 2013/115 REK Vest. A Residence Permit for the Norwegian researcher was issued by the Immigration Services Department, Tanzania.

### Data collection

#### Medical birth registry

In 2000, the KCMC established a Medical birth registry (MBR) database. The MBR maintains records information on every birth at KCMC, based on patient files and an interview with every mother during the first 24 h after childbirth [39]. This data include demographics, the mother’s health before and during pregnancy, labor characteristics, the mother’s health after childbirth up to 7 days after delivery, and the health of the newborn. For this study, we retrieved data on a birth cohort selected from MBR to investigate patient outcomes at KCMC. We verified the MBR data by the delivery books and the documentation of the admissions to the labor ward. The study population comprised all mothers with a gestational age ≥ 28 weeks. We excluded mothers whose children were born before hospital arrival. The MBR did not contain data on blood transfusions given after birth. During the study period, data on parity were not available in the MBR.

#### Power calculation

We considered whole blood transfusions the dependent variable in power calculations. We estimated that, to detect a reduction in blood transfusion rates from 3% before training to 2% after training, 1700 files would be needed in each group (alfa = 0.05, power = 80%).

#### Random samples

The rates of whole blood transfusions given after childbirth were determined by reviewing 2000 MBR files randomly selected before training and 2000 files randomly selected after training. The files were hard-copies that included admission and discharge notes, partographs, operation sheets, and blood transfusion request and report forms. The files were thoroughly checked for all administered transfusions at Department of Obstetrics and Gynecology, KCMC; whole blood transfusions and fresh frozen plasma. Mothers who had given birth within the study periods but received blood transfusions due to other conditions, were not included as being recipients of blood transfusions in the birth cohort.

MBR data on maternal deaths reflected only the first week after childbirth. In contrast, reports on maternal deaths from the Department of Obstetrics and Gynecology at KCMC included deaths from conception until 42 days after childbirth [40]. Results for maternal deaths in this study were based on the departmental reports.

### Statistical analysis

Categorical variables for patient outcomes in the MBR database and random samples were analyzed with the Chi-square test or Fisher’s exact test. Continuous variables were tested with normality plots and, when normally distributed, they were presented as the mean and standard deviation (SD). Continuous variables were compared for significant differences with the Mann–Whitney U Test. Logistic regression was performed to explore associations between whole blood transfusion (the dependent variable) and the time period of data collection; i.e., before training (2012) and after the first training (November 2013 to October 2014) as the main independent variable.

Confounders, possible risk factors, and the main factors of interest were tested one by one for their effects on the transfusion rate. These variables comprised maternal age, multiple pregnancy, birth weight, transfers from other health facilities during labor, CS, vacuum delivery, induced labor, episiotomy, preeclampsia, malaria, antepartum hemorrhage, and FGM/C. Variables with unadjusted *p*-values >0.25, variables with high frequencies of missing values, and variables associated with high residuals were not included in the final model. The final model comprised the time period of data collection, CS, transfers from other hospitals, maternal age, FGM/C, and induced labor.

The accessibility of facility equipment, medicines, and whole blood transfusion units remained constant throughout the study period.

The random selections and all analyses were conducted with IBM SPSS Statistics, Version 22.0 (IBM Corp., Armonk, NY, USA).

## Results

In October 2013, 70 staff members of a total staff of 83 employees in the Department of Obstetrics and Gynecology were trained, including 35 nurse/midwives (95%), 11 doctors (50%), and 24 medical attendants (100%). In November 2014, 67 staff members of a total staff of 87 employees were trained, including 40 nurse/midwives (100%), 7 doctors (29%), and 20 medical attendants (87%). Among the staff trained in 2013 and 2014, 17 were new employees (25%).

We identified 4095 deliveries before the training (2012) and 4031 deliveries 12 months (November 2013 to October 2014) after the first training period. The random samples comprised 2000 women in both groups. Of these randomly selected files, we were able to identify 1667 files (83%) and 1641 files (82%), respectively, based on the unique hospital number for every mother (*n* = 3308). Not all unique hospital numbers were corresponding with a mother who had given birth. Some of the files belonged to male patients, some to children, and some files were not found due to possible misplacement.

Data extracted from the random samples at KCMC, showed incidence of PPH of 0.9% before training and 1.3% after training (*p* = 0.23). However, the transfusion rate was significantly reduced. Compared to pre-training when 53 mothers received a total of 79 units of whole blood transfusions (average 1.5 unit), post-training data showed that 28 mothers received 50 units of whole blood transfusions (average 1.8 unit). The 47% reduction in number of mothers who were transfused, was statistically significant (*p* < 0.01).

We observed significant differences between groups in preeclampsia (p < 0.01), malaria (p < 0.01), induced labor (p < 0.01), and antepartum hemorrhage (*p* = 0.04), while postpartum hemorrhage based on estimated blood loss remained unchanged (Table 1). We did not observe any significant differences in maternal age, multiple pregnancy, FGM/C, and transfers from other hospitals during labor. There were significant increases in the numbers of CS and vacuum deliveries (both p < 0.01) from the pre- to the post-training periods, but the numbers of episiotomies were similar between groups. Child characteristics, like the mean gestational age and the number of infants with Apgar scores <7 after 5 min, were similar between groups, but the mean birth weight was significantly higher post-training compared to pre-training (Table 1).

**Table 1:** Patient outcomes in random samples collected before (pre-training) and after (post-training) multi-professional, scenario-based training of the maternity staff

	Pre training (n = 1667)		Post training (*n* = 1641)		p-value
Maternal and labor characteristics					
Maternal age (mean)	27.9	SD 6.5	27.9	SD 6.2	0.48
Multiple pregnancy	54	3.2%	67	4.1%	0.19*
Antepartum hemorrhage	19	1.1%	33	2%	0.04*
Preeclampsia	42	2.5%	84	5.1%	<0.01*
Malaria	128	7.7%	78	4.8%	<0.01*
Induced labor	346	20.8%	513	31.3%	<0.01*
Female genital mutilation and cutting	216	13%	192	11.7%	0.31*
Transfers from another hospital during labor	442	26.5%	455	27.7%	0.43*
Patient outcome					
Whole blood transfusions	53	3.2%	28	1.7%	<0.01*
Estimated blood loss, ml (mean)	237	SD 244	243	SD 246	0.19
Postpartum hemorrhage	15	0.9%	22	1.3%	0.23*
Cesarean section	570	34.2%	669	40.8%	<0.01*
Vacuum delivery	8	0.5%	20	1.2%	0.02*
Episiotomy	27	1.6%	19	1.2%	0.26*
Child characteristics					
Gestational age, days (mean)	282	SD 15	283	SD 13	0.46
Birth weight, grams; 1st child (mean)	3076	SD 583	3126	SD 600	0.01
Apgar score < 7 after 5 min; 1st child (n)	30	1.8%	42	2.6%	0.13*

Values represent the number (%) or the mean (SD) of the indicated birth cohort. The p-values indicate differences between medians, obtained with the Mann-Whitney U test

*The chi-square test or Fisher’s exact test was used to test for differences between proportions

The multivariable logistic regression analysis showed that whole blood transfusions were significantly associated with the time period, when controlling for number of CS, number of transfers from other hospitals, maternal age, the presence of FGM/C and labor induction (Table 2).

**Table 2:** Results of logistic regression analysis show factors significantly related to the need (no/yes) for whole blood transfusions (dependent variable; *n* = 81)

Independent variables	Unadjusted OR	(95% CI)	p-value	Adjusted OR	(95% CI)	p-value
Time periods of data collection (2012 vs. Nov 2013 to Oct 2014)	0.52	0.33–0.82	<0.01	0.45	0.28–0.73	<0.01
Total cesarean section (no/yes)	4.71	2.89–7.68	<0.01	4.16	2.51–6.89	<0.01
Transfers from another hospital during labor (no/yes)	1.96	1.25–3.01	<0.01	1.68	1.05–2.69	0.03
Maternal age (years)	1.04	1.01–1.07	0.01	1.03	1.00–1.07	0.04
Female genital mutilation and cutting (no/yes)	0.45	0.27–0.76	0.03	0.52	0.30–0.89	0.02
Induced labor (no/yes)	1.55	1.02–2.36	0.04	1.11	0.61–2.04	0.73

The total number of maternal deaths in the birth cohort was similar in both study periods. However, we observed a non-significant reduction in maternal deaths related to PPH associated with training (7 deaths before vs. 4 deaths after training; *p* = 0.35).

## Discussion

In this study, we hypothesized that implementation of multi-professional, scenario-based training could contribute to improved team efficiency in relation to PPH management. The aim was to test the hypothesis that the scenario-based training on PPH-management was followed by a reduction in whole blood transfusion rate.

The most common measurement of PPH is visual estimation of blood loss, which tends to give underestimated results [41, 42]. At KCMC, based on visual estimation, PPH was diagnosed in 0.9% of mothers before training and 1.3% after training. These figures are most likely due to underreporting, although in accordance with numerous reports on PPH-incidence of below 1% up to 6% [7, 8, 12, 14–16, 18]. However, these findings are significantly lower than a reported PPH-incidence of 10–32%, including studies from Tanzania based on estimated and measured blood loss [9, 10, 17]. While WHO reports a PPH-incidence of 2% [18], some studies from high-resource countries indicate PPH-prevalence of 5% after active management of third stage of labor and 13% after expectant management [43]. A Dutch study defining PPH as blood loss >1000 ml, found an increased PPH-incidence of 6.4% in 2013 [13]. The disparity in findings for estimated blood loss might be partly explained by the fact that visual estimation is a nonstandard measurement, which is in use globally due to the method being perceived as easy, fast and cheap [41]. However, a nonstandard measurement remains subjective.

The difference in PPH-estimates might reflect the conformity to a local estimation standard. Additionally, the perceived importance of estimated blood loss after the simulation training and its consequences for the wellbeing of mothers might be a contributing factor to the varying PPH-incidence. The estimated blood loss will most likely trigger different actions to manage PPH, depending on factors like options of treatment, perceived importance, competence and quality of teamwork.

It is possible that the focus on estimation of blood loss during simulation training, combined with increased confidence related to PPH-management, may have resulted in a lower threshold for diagnosing PPH and resulted in slightly higher estimates in our study. The visual estimation is therefore likely to have revealed more mothers with blood loss >500 ml after the simulation training, but still not reflecting the true picture due to likely underestimation and underreporting of blood loss pre- and post-training.

Blood transfusion rate was regarded a more objective and reliable indicator of blood loss, as an indirect indication of severity of PPH. We observed a statistically significant 47% reduction in whole blood transfusion rate after the training. The prevalence of blood transfusions at KCMC from 3.2 to 1.7% pre-post training corresponds with findings from a retrospective study on similar simulation training in Norway, where the prevalence of blood transfusions dropped from 3.4 to 2% after training [11]. Another Norwegian study on simulation training showed a transfusion rate pre-post training of 4.6 and 4.6% respectively [44]. The reported overall maternal transfusion rate at KCMC was according to Macheku et al. 5.7% during a period of 10 years, including antenatal hemorrhage counting for 74%, PPH for 26% of the incidents [12]. Our figures on PPH-incidence at KCMC are extremely low and the transfusion rate is low. A reduction of an already reported low blood transfusion rate can strengthen the perceived effect of the intervention.

The reduction in number of mothers receiving blood transfusions after birth at KCMC was still statistically significant when controlling for CS-rate, transfers from other health facilities, maternal age, FGM/C-rate and induction of labor. Indications for prescription of whole blood transfusions remained the same and the availability of whole blood units from KCMC Clinical Laboratory was sufficient throughout the study period. Hemoglobin level at discharge was generally not available. Because of the reductions in whole blood transfusions, it is likely that the actual blood loss after simulation training was reduced compared to the period before the training.

The training seemed to result in change of practice in the wards. A probable explanation is related to improved teamwork. This might have enabled staff members to work as teams during clinical emergencies, improved communication, providing a more efficient and higher quality of maternal care. A technical skill like bimanual uterine compression, which was trained on the birthing simulator [33], might also have made a difference. It takes a combination of knowledge, skills, caution, empathy and determination to perform bimanual compression as a life-saving procedure, because of the painful procedure imposed on the mother right after childbirth. The hands-on training on bimanual uterine compression made them practice this skill during the scenarios on PPH, and might have provided the necessary competence to carry out this procedure in clinical practice.

Logistic regression analysis showed that the reduction in whole blood transfusion rate remained significant when controlling for changes in CS rates. However, the CS rate at KCMC (34–40%) was high compared to sub-Saharan countries due to its long history of being a relatively well-equipped zonal consultant hospital, and the impact of CS on PPH therefore needs some further consideration. CS is a risk factor for PPH. In this study, we found that CS was highly associated with whole blood transfusions (unadjusted OR 4.7). However, although the overall CS rate increased between periods, the frequency of whole blood transfusions decreased. A similar pattern was found regarding preeclampsia, which was significantly increased in the birth cohort after training. Preeclampsia is regarded a risk factor for PPH [45]. A reduction in transfusion rate despite an increase in preeclampsia, might support the hypothesis that simulation training could reduce the need for blood transfusions after birth.

Our data collection did not investigate the causes for different prevalence of malaria, preeclampsia, induced labor and antepartum hemorrhage within the birth cohorts. These variables, which showed significant differences from before to after training, were tested one by one by logistic regression analyses as possible confounders. None of these variables explained the significant difference in transfusion rate pre-post training.

Hawthorne effect reflects influence of attention caused by an intervention. In order to exclude the Hawthorne effect, data collection should continue as long as there might be an attention effect, to limit the threat to internal validity. Out of 28 mothers who received whole blood transfusions the first year after training, they were equally divided throughout the year. A Hawthorne effect explaining the reduction in blood transfusions, was unlikely.

Very few studies on scenario-based training on PPH have reported significant reductions in severe blood loss after birth. In fact, some interventional studies on labor management have found significant increase in estimated blood loss and the use of oxytocin, but no change in transfusion rates [46]. Another study in Zimbabwe introduced a maternity dashboard for determining performance statistics and quality indicators, combined with training in skills and teamwork. That study reported a 34% reduction in maternal mortality, but they provided no data on transfusion rates [47, 48]. An intervention study in the US showed improved response times related to PPH management among experienced clinical teams, but they did not document changes in transfusion rates [49].

To reduce maternal mortality, the World Health Organization, UNICEF, and the United Nations Population Fund have introduced Basic and Comprehensive emergency obstetric and newborn care (BEmONC and CEmONC, respectively) to low-resource countries [50]. BEmONC focuses on training personnel in evidence-based theory and practical competencies [50]. Additionally, ALSO training was established in both high- and low-resource countries. ALSO courses have been associated with a reduction in the in-hospital maternal morbidity and mortality rates [17]. A Tanzanian study indicated that ALSO training was associated with a reduction in blood loss after vaginal births, but there was no significant change in transfusion rates [10]. Jhpiego, an affiliate of Johns Hopkins University, is strongly involved in programs for maternal and newborn health in Tanzania [51]. Jhpiego, in conjunction with Laerdal Global Health, has endeavored to provide learning materials on the prevention, identification, and treatment of PPH. They designed a training package; Helping mothers survive: Bleeding after birth (BAB) [33]. A pilot study showed that confidence on bimanual uterine compression increased after training [33]. The study, which included technical skills training with the birthing simulator, MamaNatalie® [25], was supported by the BAB learning materials. Some of the scenario-based training programs have been associated with improved clinical outcomes [52] and enhanced teamwork and communication [53]. However, only one retrospective study has documented an association between simulation training on PPH in a high-resource setting, and a reduction in transfusion rates [11]. Findings from the present study is in concert with this latter study, and indicate that multi-professional, scenario-based training with a special focus on teamwork during PPH-management, is likely to reduce excessive bleeding after birth. Moreover, the clinical significance of the training may be further illustrated by the finding that out of 21 maternal deaths in both cohorts, the number of maternal deaths related to PPH, dropped from seven to four. This reduction was not statistically significant, probably due to low statistical power, and this finding should be interpreted with caution.

### Characteristics of the training that may have contributed to positive changes

The training, which included participation of the entire maternity staff, involved the following learning aspects of scenario-based training: realistic PPH-scenarios were repeated in training, vicarious observation provided an objective learning experience, and reflective learning was encouraged through debriefings within the team. This training had a strong focus on teams, which in other studies was associated with improved teamwork [54]. Including all cadres in realistic, repeated PPH-scenarios with consecutive debriefing, is a novel approach to enhanced PPH-management. However, the attendance of doctors was low (29%) in the mandatory subsequent training, and some assumptions could be made. All doctors were allocated for ALSO-training during their 4 years of residency, and some of them were due for ALSO-training 2 months after the second simulation training. The doctors might have considered the simulation training of less importance to them compared to the nurse midwives. Additionally, the availability of doctors for training might have been reduced on the day of training due to increased workload in the department that day. At KCMC, staff had clinical experience on severe complications that largely exceeds clinical practice in high-resource countries. Their extensive experience might have made the staff very responsive to training with emphasis on teamwork.

A major strength of pedagogical approach, was the simultaneous participation of all cadres in the training. The discussions on best practices among the participants may have removed some existing intellectual and inter-relational barriers between professions, enhanced mutual understanding and promoted learning. In addition, this training was implemented after years of experience with ALSO and CEmONC training. Therefore, the knowledge and skills gained in previous training sessions may have synergized with the present training to strengthen the staff’s knowledge, skills, and attitudes.

Another strength was our contextual approach, including the use of Swahili in learning materials and debriefing sessions. These debriefings practiced during training, introduced the notion of common reflection, which may increase the probability of enhanced learning from daily events. Repetition of realistic PPH-scenarios enabled the participants to engage with the character embodied by the operator for improved performance [34]. More research is needed to determine specific characteristics of the training that may be critical for achieving improved ability to handle PPH.

### Implication of findings for practice and future research

PPH can occur after any childbirth, thus, all trained health providers must be able to prevent and manage PPH [19]. As labor complication, PPH is very suitable for scenario-based training due to recognizable signs and symptoms of blood loss, like profuse bleeding after birth due to atony or an incomplete placenta, or a mother’s complaints of dizziness. The training was carried out by local faculty, who facilitated scenarios by the use of low-fidelity equipment, and debriefed the multi-professional teams after the scenarios.

Regarding implication for practice, we believe that this training is transferable to other health facilities that aim to improve their PPH-management.

In future research, it might be feasible to use a randomized controlled trial design on multi-professional scenario-based training in countries where this kind of training is not yet widely established.

### Strengths and limitations

A main strength of this study was the sample size related to data on whole blood transfusions pre-post simulation training. The Medical Birth Registry at KCMC identified the birth cohorts and provided data on maternal and neonatal outcomes. The thorough data extraction from patient files selected by random sampling, gave us reliable data with sufficient power to demonstrate significant changes in patient outcomes pre-post training.

This study is strengthened by the contextual approach of the educational intervention, which included crucial learning aspects like realism, repetition and reflection and involved the entire maternity staff.

A limitation was our quasi-experimental study design. Due to the design, we could not determine causality, only the association to transfusion rates. A randomized controlled trial that compared groups with or without training and thereby minimized selection bias, could have provided more definite results on pre- vs. post-outcomes.

The low participation of doctors during the two training sessions was a limitation of the study. The doctors have an important role to play as team members during emergencies. Participation of 50% and 29% of the doctors respectively, limited the doctors’ possibility to learn and practice teamwork. The high attendance from the rest of the maternity staff, can only to a certain extent compensate for the lack of doctors’ attendance.

## Conclusion

This study tested the hypothesis that scenario-based training could contribute to enhanced competence on PPH-management with positive consequences on team efficiency and patient outcome. Our results showed a significant, 47% reduction in the whole blood transfusion rate after training, which supported our hypothesis. Multi-professional, scenario-based training that emphasizes repetition of realistic PPH-scenarios, reflective learning, and the involvement of all maternity staff, may contribute to reduced blood transfusion rate after birth.

## Abbreviations

ALSO: Advanced life support in obstetrics
BAB: Bleeding after birth
BEmONC: Basic emergency obstetric and newborn care
CEmONC: Comprehensive emergency obstetric and newborn care
CS: Cesarean section
FGM/C: Female genital mutilation and cutting
KCMC: Kilimanjaro Christian Medical Centre
MBR: Medical birth registry
PPH: Postpartum hemorrhage
